# Reviewed article: Santos ME, Ferreira RC, Pinheiro EL, Vieira SSA, Senna MIB. Organizational readiness for change and work process of oral health teams in Primary Health Care: an exploratory study in the initial implementation phase of MonitoraSB, Minas Gerais, Brazil, 2024. Epidemiol Serv Saude. 2025:34;e20240802

**DOI:** 10.1590/S2237-96222025v34e20240802.b

**Published:** 2025-10-20

**Authors:** Sonia Regina Cardim de Cerqueira Pestana

**Affiliations:** 1 Universidade de São Paulo, São Paulo, SP, Brazil

## Peer review B

## Questionnaire

Does the manuscript contain new and significant information to justify publication? Yes

Does the Abstract (Summary) clearly and accurately describe the content of the article? Yes

Is the problem significant and concisely stated? Yes

Are the methods described comprehensively? Yes

Are the interpretations and conclusions justified by the results? Yes

Is adequate reference made to other work in the field? Yes

## Manuscript Structure

Length of article is: Adequate

Number of tables is: Adequate

Number of figures is: Adequate

Please state any conflict(s) of interest that you have in relation to the review of this paper. None

Interest: Excellent

Quality: Excellent

Originality: Excellent

Overall: Excellent

Recommendation: Accept

## Comments

Estudo de grande importância, e acredito, ser árduo, devido muitas vezes a resistência à mudanças, principalmente nos aspectos humanos e culturais. Quando o autor coloca que o alcance de padrões de qualidade do processo de trabalho pode atuar tanto como facilitador ou como barreira e constituirá no desfecho da implementação da inovação nos serviços de saúde, considero extremamente importante essa avaliação e de que maneira os profissionais deverão entender que não se está aumentando as tarefas por eles já realizadas, por isso as diretrizes têm que estar claras e bem definidas para que se tenha uma boa adesão das equipes. Outro ponto importante que considerei nesse estudo é de que o cenário dos serviços de saúde bucal nos municípios pode não ser o real já que a participação das equipes selecionadas poderiam ter processo de trabalho mais estruturado e com maior nível de prontidão, como relatada pelo autor.

Participei das duas edições do PMAQ-CEO e entendo as diferenças em um mesmo município e o receio de alguns gestores de serem punidos ou de ter um aumento de responsabilidades, faltando a compreensão sobre os benefícios do monitoramento.

OBS: Na linha 15 da página 1, no resumo, não sei se foi a minha impressão, mas a palavra monitoramento no idioma português está com uma letra a mais.

